# Association of Nut Consumption with Cardiometabolic Risk Factors in the 2008/2009 New Zealand Adult Nutrition Survey

**DOI:** 10.3390/nu7095351

**Published:** 2015-09-08

**Authors:** Rachel C. Brown, Siew Ling Tey, Andrew R. Gray, Alexandra Chisholm, Claire Smith, Elizabeth Fleming, Winsome Parnell

**Affiliations:** 1 Department of Human Nutrition, University of Otago, PO Box 56, Dunedin 9054 New Zealand; E-Mails: alex.chisholm@otago.ac.nz (A.C.); claire.smith@otago.ac.nz (C.S.); liz.fleming@otago.ac.nz (E.F.); winsome.parnell@otago.ac.nz (W.P.); 2 Nutrition Society of New Zealand, Whanganui 4543, New Zealand; 3 Clinical Nutrition Research Centre, Singapore Institute for Clinical Sciences, A*STAR, 14 Medical Drive, #07-02, Singapore 117599, Singapore; E-Mail: siewling_tey@sics.a-star.edu.sg; 4 Department of Preventive and Social Medicine, University of Otago, PO Box 56, Dunedin 9054, New Zealand; E-Mail: andrew.gray@otago.ac.nz

**Keywords:** nut intake, population survey, cardiometabolic risk factors

## Abstract

Nut consumption has been associated with improvements in risk factors for chronic disease in populations within North America, Europe and Iran. This relationship has not been investigated in New Zealand (NZ). The associations between nut consumption and cardiometabolic risk factors among New Zealanders were examined. Data from the 24-h diet recalls of 4721 participants from the NZ Adult Nutrition Survey 2008/2009 (2008/2009 NZANS) were used to determine whole and total nut intake. Anthropometric data and blood pressure were collected, as well as blood samples analysed for total cholesterol (total-C) and HDL cholesterol (HDL-C), glycated haemoglobin (HbA1c), C-reactive protein (CRP) and folate. Participants were classified according to their five-year cardiovascular disease (CVD) risk. Both whole and total nut consumers had significantly lower weight, body mass index (BMI), waist circumference and central adiposity than non-nut consumers (all *p* ≤ 0.044). Whole blood, serum and red blood cell folate concentrations were significantly higher among whole nut consumers compared to non-whole nut consumers (all *p* ≤ 0.014), with only serum folate higher in total nut consumers compared to non-total nut consumers (*p* = 0.023). There were no significant differences for blood pressure, total-C, HDL-C and HbA1c; however, significant negative associations between total nut consumption and CVD risk category (*p* < 0.001) and CRP (*p* = 0.045) were apparent. Nut consumption was associated with more favourable body composition and a number of risk factors, which could collectively reduce chronic disease.

## 1. Introduction

Nuts are rich in cis-unsaturated fatty acids, vitamins, minerals and a number of phytochemicals, which collectively contribute to reductions in chronic disease risk, particularly cardiovascular disease (CVD), seen in both epidemiological and intervention studies [1,2,3,4,5,6]. Typically, nut consumers have lower concentrations of total cholesterol (total-C) and low-density lipoprotein cholesterol (LDL-C), with studies showing little effect on HDL cholesterol (HDL-C) and triglyceride concentrations [6]. Although recent studies have reported significant reductions in all-cause mortality among nut consumers [1,2,7,8], the effect of nut consumption on the risk of chronic disease other than CVD is less clear.

Limited epidemiologic evidence suggests that nut consumption may have beneficial effects on blood pressure, especially among those without type 2 diabetes [9] and those with hypertension who have a Body mass index (BMI) lower than 25 kg/m^2^ [10]. Two recent reviews calculated mean changes in blood pressure from over 20 intervention studies and reported significant reductions for both systolic and diastolic blood pressure following nut consumption [9,11].

Studies investigating the association between nut consumption and the risk of type 2 diabetes have produced equivocal results. Two recent meta-analyses have reported no association between nut consumption and the risk of type 2 diabetes [8,12], whereas a small, but significant reduction in the incidence of type 2 diabetes was found in another recent meta-analysis by Afshin *et al.* [13]. It has been suggested the contradictory result is likely due to the differences in study selection (e.g., one study included by Afshin *et al.* did not examine the independent effects of nuts) and a lack of adjustment for BMI in the meta-analysis by Afshin *et al.* [14]. In addition, intervention trials, which have investigated the consumption of nuts on glycaemic control, have produced mixed results [11,15].

Although nuts are energy dense, several epidemiologic studies have reported that regular nut consumers tended to be leaner than non-consumers [16,17,18,19]. In support of this finding, five clinical trials specifically designed to examine the effect of regular nut consumption on body weight have reported no weight gain or less weight gain than predicted [20,21,22,23,24].

Folate has attracted public health interest because the suboptimal status of this vitamin appears to be associated with an increased risk of several chronic diseases, such as CVD [25] and certain cancers [26]. Nuts, in particular peanuts, hazelnuts and walnuts, are relatively rich sources of folate [27]. Therefore, it is of interest to assess the folate status of nut consumers.

Nationally representative data from the United States of America (USA) showed that nut and tree nut consumption was associated with a lower BMI, waist circumference and systolic blood pressure [28,29]. Nut consumers also had a lower prevalence of hypertension, low HDL-C, abdominal obesity and high fasting glucose concentrations, four important risk factors for metabolic syndrome. A recent analysis of the Adventist Heart Study-2 found that there was a significant inverse association between the frequency of nut intake and metabolic syndrome [30]. Similar results were found in participants at high cardiovascular risk [31]. Further, a cross-sectional study in Iran reported a significant association between high nut consumption and reduced dyslipidemia [32].

To date, no research has examined the association of nut intake and risk factors for chronic disease in New Zealand or indeed anywhere in the Southern Hemisphere, where dietary patterns may differ from countries where relationships between nut consumption and a variety of risk factors for disease have previously been described [29,32]. This study aimed to compare known risk factors of chronic disease between nut consumers and non-nut consumers in a cross-sectional representative survey of the New Zealand population.

## 2. Experimental Section

### 2.1. Study Population

The 2008/2009 NZ Adult Nutrition Survey (2008/2009 NZANS) was a cross-sectional survey of 4721 New Zealander aged 15 years and over. A full description of the study design and methods is available elsewhere [33], and only a summary is included here. Participants were recruited using a three-stage process where 607 mesh blocks were selected using a probability-proportional-to-size design. A mesh block is defined as a small geographical area within NZ defined by Statistics NZ. Each mesh block contains approximately 110 people in urban areas and 60 in rural areas. After random selection of a household, random selection of a participant within the household occurred. Oversampling of Maori and Pacific people and age groups 15–18 years and 71 years and over was used to achieve adequate numbers for analysis by ethnicity and age.

Informed, written consent was obtained from each participant or from the guardian of participants aged less than 18 years prior to interviews. Ethical approval was gained from the NZ Health and Disability Multi-Region Ethics Committee (MEC/08/04/049). This study was conducted according to the guidelines laid down in the Declaration of Helsinki, and all procedures involving human subjects were approved by the NZ Health and Disability Multi-Region Ethics Committee (MEC/08/04/049).

### 2.2. Dietary Assessment

Survey data were collected at the participants’ homes by trained interviewers using computer-assisted personal interview software. An interviewer-administered multiple-pass 24-h diet recall method was used to collect quantitative information on all foods and drinks the participant consumed the previous day (from midnight to midnight). It included foods and drinks consumed both at and away from home and has been previously described [33].

### 2.3. Determination of Nut Intake

For the purpose of this study, the term ‘nuts’ includes tree nuts, peanuts and mixed nuts. Tree nuts include almonds, Brazil nuts, cashews, hazelnuts, macadamias, pecans, pine nuts, pistachios and walnuts. Chestnuts, coconut and coconut products were not included in this analysis, as their nutrient profiles differ from the aforementioned ‘nuts’. Nut intake was assessed using the 24-h diet recall data from the 2008/2009 NZANS, and total nut consumption was comprised of the following three categories:

(i) Whole nuts, including tree nuts, mixed nuts and peanuts eaten whole as part of a snack (e.g., mixed nut snacks) or as an addition to a food/meal (e.g., almonds sprinkled on a salad); (ii) consumed as nut butters, including those made from peanuts and tree nuts (e.g., peanut butter, hazelnut spread); and (iii) consumed as an ingredient of a recipe/dish or a commercial products (e.g., breakfast cereals, snack bars, satay sauce). Participants who reported consuming zero quantity of any nuts in their 24-h diet recall were classified as ‘non-nut consumers’. ‘Total nut consumers’ were participants who reported consuming any of whole nuts, nut butters and/or hidden sources of nuts. ‘Whole nut consumers’ were participants who reported consuming any amount of whole nuts.

### 2.4. Blood Collection and Analysis

Non-fasting blood samples were collected from 3348 participants at local healthcare clinics. Blood was collected into three vacutainers, two containing EDTA for plasma and one additive free for serum. Vacutainers were couriered to a central processing laboratory for analysis of total-C and HDL-C, HbA1c and CRP. Further aliquots were sent to the Department of Human Nutrition, University of Otago, for analysis of whole blood, serum and red blood cell (RBC) folate concentrations.

Serum total-C was measured enzymatically and serum HDL-C using the Ultra HDL assay, on the ARCHITECT cSystem (Abbott). Serum CRP was measured using the immunoturbidimetric assay on the Abbott. HbA1c was determined in whole blood using an ion-exchange high performance liquid chromatography method (Bio-Rad Variant II). The laboratory used for these measurements subscribes to the Royal College of Pathologists Australasia Quality Assurance Program.

Whole blood and serum folate concentrations were measured using the microbiological assay with chloramphenicol-resistant Lactobacillus casei as the test micro-organism, as described by O’Broin *et al.* [34]. RBC folate concentration was calculated using the following equation:

(1) RBC folate = whole blood folate − [serum folate × (1 − haematocrit)/haematocrit]

The accuracy of the microbiological assay was monitored using a three-level certified reference for serum folate from the National Institute of Standards Technology (NIST, USA).

### 2.5. Blood Pressure

Blood pressure was measured in triplicate using an Omron blood pressure monitor (Model HEM-907, Kyoto, Japan). There was a one-minute period between measurements. The first blood pressure reading is considered the most unreliable [35]; thus, the mean of the second and third measurements was calculated.

### 2.6. Anthropometric Measurements

Trained interviewers carried out height and weight measurements in duplicate. Standing height was measured using a stadiometer (Seca 214, Seca, Hamburg, Germany) and weight using electronic scales (Tanita HD-351, Tanita, Tokyo, Japan). BMI was calculated as weight (kg)/(height (m)^2^). The World Health Organization BMI cutoffs were used to categorise BMI status in participants aged 19 years and over. The Cole age- and sex-specific BMI cutoffs were used to categorise BMI status in those aged 15–18 years [36,37].

Waist circumference (WC) was measured at the narrowest point between the lower costal border and the top of the iliac crest. Measurements were taken over light clothing using an anthropometric tape measure (Model W606PM, Lufkin, Apex Tool Group, MD, USA). Measurements were taken to the nearest 0.1 cm.

A body shape index (ABSI) was also calculated [38]. This index, based on weight, height and waist circumference, has been developed because the strong correlation between BMI and waist circumference can make it difficult to differentiate the two as epidemiological risk factors. A body shape index is relatively uncorrelated with height, weight or BMI, while remaining positively correlated with waist circumference [38]. A body shape index has been shown to be a better predictor of mortality than waist circumference [39]. A high ABSI indicates that WC is higher than expected for a given height and weight, corresponding to a more centrally-concentrated body volume. The ABSI is calculated as follows, where waist circumference and height are expressed in meters:

(2) $$\text{ABSI}=\frac{WC}{\sqrt[{3}]{BMI^{2}}\sqrt{height}}$$

### 2.7. Cardiovascular Disease Risk

Cardiovascular disease risk was calculated for participants aged 35–74 years (*n* = 1623) using the NZ adapted Framingham Cardiovascular Risk charts [40]. These charts categorise 5-year cardiovascular disease risk (fatal and non-fatal) into the following 8 categories: <2.5%, 2.5%–5% and 5%–10% (mild risk); 10%–15% (moderate risk); 15%–20% (high risk); 20%–25%, 25%–30%; and >30% (very high risk). Risk assessment is based on sex, age, total-C:HDL-C ratio, systolic blood pressure, smoking status and the presence/absence of diabetes. Maori, Pacific and Indo-Asian (Indian, including Fijian Indian, Sri Lankan, Afghani, Bangladeshi, Nepalese, Pakistani and Tibetan) participants are moved up one risk category as their risk of CVD may be underestimated using these charts [41].

### 2.8. Health Risk Factor Cutoffs

Participants were categorised based on being overweight or obese (BMI ≥25 kg/m^2^); having abdominal obesity (waist circumference ≥102 cm for males and ≥88 cm for females); having hypertension (SBP ≥130 mmHg or DBP ≥85 mmHg; or having low HDL-C (≤1.03 mmol/L for males and ≤1.29 mmol/L for females).

Participants with diabetes were defined as those who self-reported doctor-diagnosed diabetes or those who had an HbA1c ≥6.5% (48 mmol/mol) [42]. Participants with pre-diabetes included those who had an HbA1c between 5.7% (39 mmol/mol) and 6.4% (46 mmol/mol) inclusive and who did not self-report doctor diagnosed diabetes.

### 2.9. Demographic Variables

Demographic variables were selected *a priori* after reviewing the literature. Variables included sex, age category (15–18, 19–30, 31–50, 51–70, 71+ years), prioritised ethnicity, NZ Index of Deprivation (NZDep06) and education. Information was also collected during the interview on smoking status (never smoker, ex-smoker, current smoker) and use of statins.

#### 2.9.1. Ethnicity

Self-reported ethnicity was categorised into one of three ethnic groups based on a priority classification system using the coding prioritisation order (from highest to lowest) of Maori, Pacific and New Zealand European and other (NZEO). For example, if a participant identified as both Maori and NZ European, they were classified as Maori.

#### 2.9.2. New Zealand Index of Deprivation (NZDep06)

NZDep06 is an area-based measure of deprivation, which uses nine variables from the NZ Census reflecting specified dimensions of both material and social deprivation. Each mesh block in NZ is given a score between 1 and 10, with a score of 1 reflecting the least deprived areas and 10 the most deprived. For the purpose of the 2008/2009 NZANS, these scores were divided into quintiles where Quintile 1 represents the 20% least deprived and Quintile 5 the 20% most deprived areas.

#### 2.9.3. Education

Participants were asked to report their highest school level qualification and, where appropriate, their highest post-school qualification. Three groups, comprising no formal school qualification, secondary school qualification only or post-school qualification (including trade certificates and university degrees), were derived for these analyses.

### 2.10. Statistical Analysis

The complex survey design described above was accounted for in all analyses presented here. This included incorporating both weights and clustering. The weights used were post-stratification weights for the questionnaire component of the NZANS (when comparing reported nut consumption between demographic groups and comparing anthropometric outcomes between nut consumption groups) and post-stratification weights for the blood component of the NZANS (comparing biochemical outcomes between nut consumption groups) and are intended to reflect the NZ population aged 15 years and above. Stata’s default method for calculating survey-adjusted standard errors (Taylor linearization) was used for all analyses.

Log-transformations were made where this improved residual normality and/or homoscedasticity. Variables, which were log-transformed, include weight, BMI, waist circumference, total cholesterol, HDL-cholesterol, total:HDL-C ratio, C-reactive protein, HbA1c, whole blood folate, serum folate and red blood cell folate. These variables are presented as geometric means with differences reported as the percentage difference between the geometric means. Unadjusted and adjusted differences for outcomes between nut consumers and non-nut consumers are presented. Survey regression models, including sex, age group, prioritised ethnicity, NZDep06 quintile and education (blood pressure was further adjusted for smoking status and BMI category; all blood variables were further adjusted for BMI category; and CRP was further adjusted for smoking status), were used to calculate adjusted differences in outcomes between nut consumers and non-nut consumers. Survey logistic regression was used to estimate the adjusted odds ratios (OR) and 95% confidence intervals (CIs), for overweight/obese, abdominal obesity, hypertension, low HDL-C, diabetes and pre-diabetes, with the following variables entered into the model: sex, age, NZDep06, education, ethnicity and BMI. Ordinal logistic regression, including NZDep06 quintile, education and BMI category, was used to estimate the adjusted-for (Maori, Pacific and Indo-Asian ethnicities) CVD risk category. Proportionality was examined using generalised ordinal logistic regression without adjustment for the complex survey design to see if there was evidence against proportionality at the variable level or overall. Standard regression diagnostics were used in all cases.

Stata Statistical Software 12.1 (Statacorp LP, College Station TX, USA) was used for all statistical analyses. All statistical tests were two-sided; *p* < 0.05 was considered statistically significant, and 0.05 ≤ *p* < 0.10 is noted as a non-significant tendency, where these may suggest areas for further research or support the interpretation of other results. As this study is exploratory, no formal adjustment for multiple comparisons was made, and marginally significant results should be interpreted with caution.

## 3. Results

### 3.1. Characteristics of the Sample

Table 1 describes the characteristics of the 2008/2009 NZANS sample. A total of 4721 participants were recruited and completed a 24-h diet recall.

**Table 1:** Characteristics of survey participants.

	All Survey Participants	
Demographic	*n*	survey weighted %
Total population	4721	
Sex		
Male	2066	48.6
Female	2655	51.4
Age		
15–18 years	699	7.0
19–30 years	718	19.7
31–50 years	1344	36.7
51–70 years	895	27.1
71+ years	1065	9.6
Ethnicity		
NZEO ^a^	2980	84.3
Maori	1040	11.1
Pacific	701	4.6
NZDep06 quintile ^b^		
Q1 (least deprived)	664	20.2
Q2	829	21.4
Q3	761	21.3
Q4	1072	19.0
Q5 (most deprived)	1395	18.1
Highest educational qualification		
No school qualification	1217	18.1
School	1413	26.5
Post-school	2057	55.4
Body mass index (kg/m^2^)		
<25	1409	34.9
25–29.9	1581	37.1
≥30	1513	28.0
Smoking status		
Never smoked	2393	50.8
Ex-smoker	1274	26.5
Current smoker	1074	22.8

^a^ New Zealand European and other; ^b^ New Zealand Index of Deprivation.

### 3.2. Nut Intake

The nut intakes of New Zealanders have been described previously [43]. In brief, the percentage of the population consuming whole nuts (a subset of total nuts) and nuts from all sources (total nuts, including whole nuts, nut butters and nuts from hidden sources) on the day of their 24-h diet recall were 6.9% and 28.9%, respectively. The mean daily intake among the whole population was 2.8 g and 5.2 g for whole and total nuts, respectively. Among nut consumers only, the mean daily intake was 40.3 g for whole nuts and 17.9 g for total nuts (which included whole nuts, nut butters and nuts from hidden sources). In terms of prevalence, there were no significant differences between males and females.

### 3.3. Anthropometric Measurements

In the fully-adjusted model, body weight, BMI, waist circumference and ABSI were significantly lower among whole nut consumers (a subset of total nut consumers) compared to non-whole nut consumers (all *p* ≤ 0.029) and for total nut consumers (including whole nut, nut butter and nuts from hidden source consumers) compared to non-total nut consumers (all *p* ≤ 0.044) (Table 2).

**Table 2:** Mean (95% CI) body composition and blood pressure among nut and non-nut consumers.

	Total *n*	Non-Nut Consumers	*n*	Nut Consumers	*n*	Unadjusted Difference	Unadjusted *p*-Value	Adjusted Difference *	Adjusted *p*-Value
*Whole nuts*									
Weight (kg)	4519	77.0 (76.2, 77.8)	4288	73.5 (70.7, 76.5)	231	−4.5 (−8.2, −0.5)	0.026	−4.0 (−7.1, −07)	0.017
Body mass index (kg/m^2^)	4503	27.1 (26.8, 27.3)	4272	26.1 (25.3, 26.9)	231	−3.6 (−6.6, −0.6)	0.019	−3.9 (−6.7, −1.0)	0.008
Waist circumference (cm)	4519	90.5 (89.8, 91.2)	4288	87.3 (84.9, 89.8)	231	−3.5 (−6.3, −0.6)	0.018	−3.7 (−6.1, −1.3)	0.003
ABSI	4459	0.0766 (0.0763, 0.0768)	4229	0.0759 (0.0750, 0.0768)	230	−0.0007 (−0.0016, 0.0028)	0.165	−0.0009 (−0.0017, <−0.0001)	0.029
Systolic blood pressure (mmHg)	4632	120.3 (118.9, 121.7)	4396	120.3 (115.9, 124.6)	236	<0.1 (−4.5, 4.5)	1.000	−1.4 (−5.4, 2.6)	0.497
Diastolic blood pressure (mmHg)	4632	70.7 (69.9, 71.6)	4396	70.7 (68.0, 73.5)	236	<0.1 (−2.9, 2.9)	1.000	−0.8 (−3.4, 1.8)	0.553
*Total nuts*									
Weight (kg)	4519	77.5 (76.6, 78.4)	3383	75.0 (73.6, 76.3)	1136	−3.3 (−5.4, −1.1)	0.003	−2.0 (−4.0, −0.1)	0.044
Body mass index (kg/m^2^)	4503	27.3 (27.0, 27.6)	3370	26.4 (26.0, 26.8)	1133	−3.1 (−4.9, −1.3)	0.001	−2.3 (−4.0, −0.5)	0.012
Waist circumference (cm)	4519	91.2 (90.4, 92.0)	3383	88.1 (86.8−89.3)	1136	−3.4 (−5.0, −1.8)	<0.001	−2.5 (−3.9, −1.0)	0.001
ABSI	4459	0.0768 (0.0766, 0.0772)	3338	0.0757 (0.0752, 0.0761)	1121	−0.0012 (−0.0017, −0.0007)	<0.001	−0.0009 (−0.0013, −0.0005)	<0.001
Systolic blood pressure (mmHg)	4632	122.3 (120.8, 123.8)	3479	120.5 (118.6, 122.4)	1153	−1.4 (−3.9, 1.2)	0.289	−1.3 (−3.5, 1.0)	0.263
Diastolic blood pressure (mmHg)	4632	72.0 (71.0, 73.0)	3479	70.8 (69.6, 72.0)	1153	−0.9 (−2.5, 0.6)	0.237	−1.1 (−2.6, 0.3)	0.132

* All variables adjusted for sex, age, NZDep, education level and ethnicity; blood pressure further adjusted for smoking status and BMI; blood pressure and ABSI are presented as arithmetic means; weight, BMI and waist circumference were log transformed, and geometric means are presented, with differences reported as the percentage difference between geometric means; ABSI = a body shape index; the ABSI is calculated as follows, where waist circumference (WC) and height are expressed in metres: ABSI = WC/BMI^2/3^height^1/2^.

### 3.4. Blood Pressure

Neither systolic nor diastolic blood pressure differed significantly between whole nut consumers (a subset of total nut consumers) and non-whole nut consumers (both *p* ≥ 0.497) (Table 2), nor did they differ between total nut consumers (including whole nut, nut butter and nuts from hidden source consumers) and non-total nut consumers (both *p* ≥ 0.132).

### 3.5. Biochemical Outcomes

There was a non-significant tendency for a lower total-C:HDL-C ratio in whole nut consumers (a subset of total nut consumers) compared to non-whole nut consumers (*p* = 0.090), with significantly higher HDL-C (*p* = 0.024) and lower total-C:HDL-C ratio (*p* = 0.036) among total nut consumers (including whole nut, nut butter and nuts from hidden source consumers) compared to non-total nut consumers before adjustment of potential confounders. However, after adjustment, total cholesterol, HDL-C and total-C:HDL-C ratio did not differ significantly between whole nut consumers and non-whole nut consumers (all *p* ≥ 0.147) nor between total nut consumers and non-total nut consumers (all *p* ≥ 0.310) (Table 3).

There was a non-significant tendency for lower CRP among whole nut consumers (*p* = 0.078), but this was no longer evident after adjustment for potential confounders (*p* = 0.451); whereas among total nut consumers, CRP was significantly lower than non-total nut consumers for both the unadjusted (*p* < 0.001) and the adjusted model (*p* = 0.045).

There was no significant difference for HbA1c between whole nut consumers and non-whole nut consumers (*p* = 0.646). Glycated haemoglobin was significantly lower for total nut consumers compared to non-total nut consumer (*p* = 0.021), but this difference was no longer evident after adjusting for potential confounders (*p* = 0.475).

After adjustment for potential confounders, whole blood folate (*p* = 0.004), serum blood folate (*p* = 0.001) and red blood cell folate (*p* = 0.014) were statistically significantly higher among whole nut consumers compared to non-whole nut consumers. In contrast, for total nut consumers, only serum folate concentration was significantly higher compared to non-total nut consumers (*p* = 0.023).

**Table 3:** Mean (95% CI) for biochemical indices for nut consumers and non-nut consumers.

Biochemical Indices	Total *n*	Non-consumers	*n*	Nut Consumers	*n*	Unadjusted Difference	Unadjusted *p*-Value	Adjusted Difference *	Adjusted *p*-Value *
*Whole nuts*									
Total cholesterol (mmol/L)	3309	5.02 (4.97, 5.08)	3108	4.99 (4.77, 5.02)	201	−0.8 (−5.1, 3.8)	0.740	−3.2 (−7.5, 1.2)	0.147
HDL-cholesterol (mmol/L)	3309	1.33 (1.31, 1.34)	3108	1.38 (1.31, 1.44)	201	3.8 (−1.1, 9.0)	0.131	−1.0 (−4.9, 3.0)	0.611
Total-C:HDL-C ratio	3309	3.79 (3.73, 3.85)	3108	3.62 (3.44, 3.81)	201	−4.4 (−9.3, 0.7)	0.090	−2.2 (−6.4, −2.2)	0.318
C-reactive protein (mg/L)	3310	1.60 (1.53, 1.68)	3109	1.39 (120, 1.61)	201	−13.0 (−25.6, 1.6)	0.078	−5.5 (−18.5, 9.5)	0.451
HbA1c (%)	3348	5.53 (5.50, 5.56)	3147	5.49 (5.39, 5.60)	201	−0.7 (−2.6, 1.3)	0.488	−0.4 (−2.1, 1.3)	0.646
Whole blood folate (nmol/L)	2929	351 (342, 360)	2749	409 (377, 444)	180	16.6 (7.0, 27.0)	**<0.001**	13.0 (4.0, 22.8)	**0.004**
Serum folate (nmol/L)	3277	22.9 (22.1, 23.6)	3076	28.8 (25.9, 32.1)	201	26.2 (12.9, 41.1)	**<0.001**	19.7 (7.6, 33.1)	**0.001**
Red blood cell folate (nmol/L)	2821	800 (780, 821)	2646	928 (853, 1,009)	175	15.9 (6.4, 26.3)	**0.001**	11.6 (2.6, 21.7)	**0.014**
*Total nuts*									
Total cholesterol (mmol/L)	3309	5.03 (4.96, 5.09)	2426	5.01 (4.96, 5.11)	883	−0.3 (−2.6, 2.0)	0.778	−1.1 (−3.2, 1.0)	0.310
HDL-cholesterol (mmol/L)	3309	1.32 (1.30, 1.34)	2426	1.36 (1.33, 1.38)	883	3.0 (0.4, 5.7)	**0.024**	−0.3 (−2.5, 1.9)	0.781
Total-C:HDL-C ratio	3309	3.81 (3.75, 3.88)	2426	3.69 (3.59, 3.79)	883	−3.2 (−6.2, −0.2)	**0.036**	−0.8 (−3.5, 1.9)	0.553
C-reactive protein (mg/L)	3310	1.67 (1.58, 1.76)	2427	1.41 (1.30, 1.52)	883	−15.8 (−22.9, −8.0)	**<0.001**	−7.6 (−14.4, −0.2)	**0.045**
HbA1c (%)	3348	5.55 (5.51, 5.58)	2456	5.48 (5.43, 5.53)	892	−1.2 (−2.2, −0.2)	**0.021**	−0.3 (−1.3, 0.1)	0.475
Whole blood folate (nmol/L)	2929	354 (344, 364)	2161	359 (341, 377)	768	1.3 (−4.3, 7.4)	0.643	0.3 (−5.0, 6.0)	0.903
Serum folate (nmol/L)	3277	22.4 (21.6, 23.3)	2395	25.3 (23.8, 26.8)	882	12.7 (5.1, 20.8)	**0.001**	8.4 (1.1, 16.1)	**0.023**
Red blood cell folate (nmol/L)	2821	808 (785, 831)	2071	815 (774, 857)	750	0.9 (−4.8, 6.9)	0.773	−0.6 (−6.0, 5.1)	0.831

* All variables adjusted for sex, age, NZDep, education level and ethnicity; CRP was further adjusted for smoking status; total cholesterol, HDL cholesterol and Total:HDL-C ratio were further adjusted for smoking status, cholesterol-lowering medication and percent energy from saturated fat; all variables were log-transformed, and geometric means are presented, with differences reported as the percentage difference between geometric means.

### 3.6. Cardiovascular Disease Risk

There was evidence of non-proportionality for some of the covariates, but not for the nut consumption variables. In particular, for the whole nut consumption (a subset of total nut consumption) model, there was evidence of non-proportionality for the highest levels of deprivation and education and for the total nut consumption (including whole nut, nut butter and nuts from hidden source consumption) model; there was evidence of non-proportionality for the highest level of deprivation. Modelling these did not materially change the odds ratios for nut consumption (non-survey adjusted odds ratios for the nut consumption variables were identical to two decimal places in both cases, and the interpretation of covariates was similar in both cases), and so, survey-adjusted ordinal logistic regression models assuming proportionality are presented below. There was no evidence of an association between whole nut consumption and CVD risk category (proportional odds ratio = 0.83; 95% CI: 0.58–1.19; *p* = 0.321). However, there was a significant negative association between total nut consumption and CVD risk category (proportional odds ratio = 0.61; 95% CI: 0.47–0.80; *p* < 0.001).

In both models, living in the most deprived areas, having a lower education level and having a higher BMI were all association with increased CVD risk (all *p* ≤ 0.005).

### 3.7. Odds Ratios for Health Risk Factors

The odds of being overweight or obese were 40% lower for whole nut consumers (a subset of total nut consumers) compared to non-whole nut consumers (*p* = 0.015) and 36% lower for total nut consumers (including whole nut, nut butter and nuts from hidden source consumers) (*p* = 0.020) compared to non-total nut consumers (Table 4). Similarly, the odds of having abdominal obesity were 46% lower (*p* = 0.012) in whole nut consumers and 32% lower (*p* = 0.004) in consumers of all nuts. Consumers of whole nuts had 57% lower odds of pre-diabetes (*p* = 0.004). There was no significant difference in the odds of pre-diabetes or diabetes between consumers of all nuts and non-nut consumers (both *p* ≥ 0.346).

There were no significant differences in the odds of having low HDL-C or hypertension for whole and total nut consumers compared to non-consumers (all *p* ≥ 0.448).

**Table 4:** Odds ratio (95% CI) for diabetes and risk factors for chronic disease by nut consumption.

	Unadjusted Odds ratio	Unadjusted *p*-Value	Adjusted Odds ratio *	Adjusted *p*-Value †
*Whole nuts*				
Overweight or obese	0.66 (0.44, 0.99)	0.045	0.60 (0.40, 0.90)	0.015
Abdominal obesity	0.57 (0.37, 0.89)	0.012	0.54 (0.34, 0.87)	0.012
Hypertension	0.91 (0.61, 1.38)	0.682	0.85 (0.53, 1.36)	0.502
Low HDL-C	0.77 (0.49, 1.22)	0.268	0.97 (0.61, 1.55)	0.904
Diabetes	0.91 (0.45, 1.81)	0.780	1.05 (0.49, 2.26)	0.898
Pre-diabetes	0.52 (0.31, 0.88)	0.016	0.43 (0.34, 0.76)	0.004
*Total nuts*				
Overweight or obese	0.72 (0.57, 0.91)	0.006	0.74 (0.58, 0.95)	0.020
Abdominal obesity	0.66 (0.52, 0.84)	0.001	0.68 (0.52, 0.88)	0.004
Hypertension	0.83 (0.65, 1.04)	0.116	0.90 (0.69, 1.19)	0.456
Low HDL-C	0.78 (0.61, 0.99)	0.042	0.90 (0.70, 1.17)	0.448
Diabetes	0.63 (0.42, 0.96)	0.031	0.80 (0.51, 1.27)	0.346
Pre-diabetes	0.80 (0.63, 1.03)	0.082	0.88 (0.67, 1.16)	0.360

* Calculated using survey multiple logistic regression and adjusted for sex, age, NZDep06, education, ethnicity and BMI (except for overweight/obesity and abdominal obesity, which were not adjusted for BMI); **†** overall *p*-value from regression models; overweight or obesity, BMI ≥25 kg/m^2^; abdominal obesity, waist circumference ≥102 cm for males and ≥88 cm for females; hypertension, systolic BP ≥130 mmHg or diastolic BP ≥85 mmHg; low HDL-C, ≤1.03 mmol/L for males and ≤1.29 mmol/L for females; pre-diabetes included those with an HbA1c result between 5.7% (39 mmol/mol) and 6.4% (46 mmol/mol) inclusive and did not self-report doctor diagnosed diabetes; diabetes included those who self-reported doctor-diagnosed diabetes or those who had an HbA1c ≥6.5% (48 mmol/mol).

## 4. Discussion

This is the first study to assess the association between nut consumption and risk factors for chronic disease, in a cross-sectional survey of a population in NZ. This study confirms the results from large cross-sectional surveys undertaken in the Northern Hemisphere [29,30,31,32], which report that nut consumption is associated with better outcomes for a number of risk factors for chronic disease. Body weight, BMI and measures of central adiposity were significantly lower among nut consumers compared to non-nut consumers, as was the risk of being overweight or obese. In addition, CRP, a marker of inflammation, was significantly lower among total nut consumers compared to non-nut consumers. Furthermore, whole blood, serum and red blood cell folate were significantly higher among whole nut consumers compared to non-whole nut consumers. Collectively, these differences are likely to confer long-term beneficial health effects among regular nut consumers.

Although there were some significant differences between total-C and the total-C:HDL-C ratio between nut consumers and non-nut consumers, these disappeared after adjustment for potential confounders. This is in contrast to the majority of epidemiologic studies and randomised controlled trials, which show better lipid profiles or improvements in blood lipids and lipoproteins with the inclusion of nuts in the regular diet. A meta-analysis of 25 clinical trials reported dose-response reductions in total-C and LDL-C, with no effect on HDL-C, and a reduction in triglycerides [6]. However, the findings of the present study in regards to blood lipoproteins are not unique. A cross-sectional analysis of the Prevencion con Dieta Mediterranea (PREDIMED) study also found no evidence of an association between nut consumption and dyslipidemia [31]. Furthermore, O’Neil *et al.* failed to show a difference in total cholesterol between ‘out of hand nut’ consumers and non-consumers in a representative sample from the USA [44]. Out of hand nut consumers were defined as those who ate nuts solely as nuts, not as components of other food products. This is similar to the definition for whole nut consumers used in the present study. It has been suggested that these consumers may differ from consumers of nuts from all sources in that they make a conscious decision to eat nuts. Conversely, analysis of National Health and Nutrition Examination Survey (NHANES) data 1999–2004 reported significantly higher concentrations of HDL-C among nut consumers compared to non-consumers [29]. In addition, Askari *et al.* reported that nut consumption was associated with a reduction in LDL-C, triglycerides and apoB:apoA ratio, with total-C significantly reduced among female participants only, in an Iranian cohort [32].

The blood samples collected in the present study were not collected in a fasting state; therefore, LDL-C and triglycerides were unable to be measured. A meta-analysis has suggested that reductions in total and LDL-C are blunted in those who are obese [6]. One possible explanation for the lack of association between cholesterol concentrations and nut consumption observed in this study could be the high rates of obesity in the NZ population, where over 36% are classified as overweight and 28% as obese [45].

Despite the lack of difference in these specific lipoproteins, there was a significant negative association between total nut consumption and CVD risk category. The calculation of the CVD risk category takes into account the total-C:HDL-C ratio, as well as sex, age, systolic blood pressure and smoking and diabetes status.

A more consistent finding across different populations is the significantly lower body weight, BMI and waist circumference observed among nut consumers [46,47]. In agreement with other investigators [31,44,48,49], this study found a lower risk of being overweight and obesity among nut consumers, despite the fact that nut consumers report higher energy intakes compared to non-nut consumers. This finding is consistent with other epidemiological studies [16,17,18,19], which show that nut consumers tend to be leaner than non-nut consumers, and clinical trials [20,21,22,23,24], which report that when nuts are added to the diet, there is no weight gain or less weight gained than predicted. There are several mechanisms that may explain this consistent finding. Firstly, the composition of nuts, which contain protein and fibre, may result in dietary compensation. Indeed, a recent review estimated that dietary compensation accounted for 65%–75% of the additional energy provided by nuts [50]. Recent studies have also suggested that a substantial proportion of the energy in nuts is lost in the faeces, suggesting that the metabolisable energy from nuts is 9%–32% less than that predicted using the Atwater factors [51,52,53]. A third explanation is possibly the increase in metabolic rate observed with the higher intake of unsaturated fats, although this has only been reported in studies investigating peanuts [20,54,55].

There were no significant differences in blood pressure between nut and non-nut consumers in the present study. Data from NHANES 1999–2004 revealed that nut consumers had significantly lower systolic blood pressure and prevalence of hypertension [29]. However, most clinical trials investigating the effects of nut consumption on blood pressure have small sample sizes and tend to show mixed results, with the majority reporting no effect on blood pressure [11,56].

Both epidemiological studies and clinical trials have also produced mixed results regarding the relationship between nut consumption and the risk of developing type 2 diabetes. Analysis of NHANES data showed lower prevalence of risk factors associated with metabolic syndrome among nut consumers [29]. In a cross-sectional analysis of the PREDIMED study in a group of individuals with a wide range of nut intake and at high risk of cardiovascular disease, nut consumption was associated with a significant reduction in the risk of obesity, metabolic syndrome and diabetes [31]. However, there were no significant associations for the components of metabolic syndrome, including high blood pressure, dyslipidemia and fasting hyperglycaemia. Collectively, the evidence suggests no association between diabetes risk and nut consumption, which is in agreement with the present study, where there was no difference in the risk of diabetes between nut and non-nut consumers. The risk of pre-diabetes was significantly reduced only among whole nut consumers compared to whole non-nut consumers. This may reflect the more healthful effects of nuts when consumed alone rather than as components of other foods. Given the equivocal findings on the association of nut consumption and diabetes and metabolic syndrome, more well-designed studies are required in order to draw definitive conclusions.

C-reactive protein is a marker of inflammation, which has been positively correlated with CVD [57]. In the present study, CRP was significantly lower among total nut consumers compared to non-total nut consumers. O’Neil *et al.* reported that ‘out of hand’ nut consumers had significantly lower CRP compared to non-nut consumers [44]. Only around one-quarter of intervention studies in this area have reported significant reductions in CRP with the regular consumption of nuts [58,59,60]. A limitation of the current study is that only CRP was measured, not high sensitivity CRP (hsCRP), which is a more sensitive measure.

Whole blood, serum and red blood cell folate concentrations were higher among whole nut consumers, whereas only serum folate was higher among total nut consumers. This finding is consistent with those of O’Neil *et al.*, who reported significantly higher serum and red blood cell folate among nut consumers in the NHANES 1999–2004 dataset [29]. Folate is also present in fruit and vegetables, and many breakfast cereals are fortified with folic acid. Therefore, these higher concentrations of blood folate could be due to the intake of nuts, but may also be a marker of a healthier diet.

The results of the present study should be interpreted with several limitations in mind. Firstly, the cross-sectional design of the study means causal inferences cannot be drawn. The improvements in risk factors observed among nut consumers in this study and others may be due to the addition of nutrient dense nuts to the diet. An alternative explanation is that nut consumption may be a marker of a healthier diet. Nut consumers may simply be more health conscious than non-consumers. Thus, a healthier lifestyle may explain the association of nuts with risk factors (*i.e*., the associations observed could be the result of residual confounding by health consciousness). This explanation cannot be excluded in the present study. A further limitation was that dietary intake relied on memory and included only one 24-h diet recall for the identification of nut consumers, so it may not represent usual nut intake. Therefore, the effects of regular nut consumption on the risk factors examined here may have been under- or over-estimated. In addition, blood samples were not fasting in order to enhance compliance. Thus, LDL-C and triglycerides were unable to be measured. Furthermore, CRP was measured, which is less sensitive than hsCRP. Waist circumference was measured over light clothing, and this is known to introduce bias to the measurement [61]. Lastly, individuals with type 1 and 2 diabetes could not be differentiated; however, over 90% of people with diabetes in NZ are reported to have type 2 diabetes [62].

The strengths of this study include the rigorous coding of food items collected through a multi-stage process and the use of NZ-specific compositional data, allowing confidence in the collected estimates of the intake for nuts. Other strengths of the study are its large sample size, permitting precise estimation of the effects, and the use of a representative, after weighting, population-based sample. In addition, several unique outcomes in relation to nut consumption were investigated, including ABSI and cardiovascular risk category.

## 5. Conclusions

In summary, this is the first study using national data from NZ to examine the effects of nut consumption on risk factors for chronic disease. In agreement with other studies conducted in the U.S., Europe and Iran, nut consumers were leaner with reduced central adiposity and had better outcomes for a variety of biochemical indices compared to non-consumers, which collectively may reduce the risk of chronic disease.
